# Genomic and comparative analysis of the T cell receptor gamma locus in two *Equus* species

**DOI:** 10.3389/fimmu.2023.1264949

**Published:** 2023-09-15

**Authors:** Serafina Massari, Francesco Giannico, Nunzia Valentina Paolillo, Angela Pala, Anna Caputi Jambrenghi, Rachele Antonacci

**Affiliations:** ^1^ Department of Biological and Environmental Science and Technologies, University of Salento, Lecce, Italy; ^2^ Department of Veterinary Medicine, University of Bari “Aldo Moro”, Bari, Italy; ^3^ Department of Biosciences, Biotechnologies and Environment, University of Bari “Aldo Moro”, Bari, Italy; ^4^ Department of Soil, Plant and Food Science, University of Bari “Aldo Moro”, Bari, Italy

**Keywords:** gamma-delta T-cell, TRG locus, TRG genes, Perissodactyla, Equus, equid genome, immunogenomics, evolution

## Abstract

The genus *Equus* is the only extant genus of the Equidae family, which belongs to Perissodactyla, an order of mammals characterized by an odd number of toes (odd-toes ungulates). Taking advantage of the latest release of the genome assembly, we studied, for the first time in two organisms belonging to the *Equus* genus, the horse (*Equus caballus*) and the donkey (*Equus asinus*), the T cell receptor gamma (TRG) locus encoding the gamma chain of the γδ T cell receptor. Forty-five Variable (TRGV) genes belonging to the seven IMGT-NC validated mammalian TRGV subgroups, 25 Joining (TRGJ) and 17 Constant (TRGC) genes organized in 17 V-J-(J)-C cassettes, in tandem on about 1100 Kb, characterize the horse TRG locus, making the horse TRG locus the one with the greatest extension and with a significantly higher number of genes than the orthologous loci of the other mammalian species. A clonotype analysis of an RNA-seq transcriptomic dataset derived from spleen of an adult healthy horse, using the complete set of the horse TRGJ germline gene sequences as a probe, revealed that, in addition to the most prominent V-J rearrangements within each cassette, there is a relevant proportion of trans-cassette V-J recombination, whereby the same TRGV genes can recombine with different TRGJ genes spliced to the corresponding TRGC genes. This recombinant event strongly contributes to the diversity of the γ chain repertoire. In the donkey TRG locus, 34 TRGV, 21 TRGJ and 14 TRGC genes distributed in 14 V-J-(J)-C cassettes were found in a region of approximately 860 kb. Although the donkey’s TRG is smaller than that of the horse, in *Equus* genus, this is still the second largest locus so far found in any mammalian species. Finally, the comparative analysis highlighted differences in size and gene content between the horse and donkey TRG loci, despite belonging to the same genus, indicating a good level of diversification within *Equus*. These data is in agreement with the evolutionary idea of the existence of a *Equus* recent common ancestor in rapid evolution, for which a mutation rate between horses and donkeys is more comparable to that between species belonging to different genera rather than to species of the same genus.

## 1 Introduction

The antigen receptors of the adaptive immune response of the vertebrates with jaws (Gnathostomata) comprise the immunoglobulins (IG) or antibodies and the T cell receptors (TR) (1). The TR is heterodimeric and consists of an α chain and a β chain, or a γ chain and a δ chain. Each chain comprises a variable (V) domain, or V-(D)-J region, and a constant (C) region encoded by multigene families arranged in a TR locus (2). The V domain has three complementarity-determining regions or CDRs (CDR1, CDR2 and CDR3). If CDR1 and CDR2 are encoded by the germline gene sequences, the CDR3 is created when the TR genomic loci undergo somatic rearrangement between genes during the development of T lymphocytes in the thymus (3–5). For α (TRA) and γ (TRG) chain loci, recombination occurs between Variable (V) and Joining (J) genes, whereas, for δ (TRD) and β (TRB) chain loci, a Diversity (D) gene is also included between a V and J gene.

Hence, the number of V, D and J genes, in the germline DNA and the randomness of the rearrangement event are primarily determinant for the diversity and the extent of the TR repertoire. Moreover, at the junctions of the rearranged genes, further diversity is generated through the deletion of germline-encoded bases and the addition of random non-templated bases, making the CDR3 the most variable portion, which contributes strongly to the antigen-binding specificity of TR (1, 2).

During transcription, mRNAs encoding protein chains are generated, and the V-(D)-J region of the V-domain is spliced to the relevant C gene encoding the C-region.

In general, the gene organization of TR loci varies among species. However, the most evident structural differences mainly concern the TRG locus (6).

Among the loci of the antigen receptors, The *Homo sapiens* TRG locus is a paradigm as it was the first complete locus of the adaptive immune response to be entered in databases as “genes” as well as conventional genes, leading in 1989 to the creation of IMGT and to immunoinformatics, a new science at the interface between immunogenetics and bioinformatics (1). The *Homo sapiens* TRG locus is located, in reverse orientation (REV), on the short arm of chromosome 7 at 7p14, and spans 160 kb (2, 7–9). The locus consists of 12-15 TRGV genes upstream of a duplicated J-C cluster, which comprises in the first part three TRGJ genes and the TRGC1 gene, and in the second part, two TRGJ genes and the TRGC2 gene (10–13). The TRGV genes belong to six different subgroups based on the absence of cross-hybridization between them and defined, in terms of sequences, by less than 75% identity at the nucleotide level in their V regions (14–16). TRGV9, expressed in 80–95% of the human peripheral T cells, is the unique member of subgroup 2 (14). TRGV10 and TRGV11, the single members of subgroups 3 and 4, respectively, have been found to be rearranged and transcribed, but they are open reading frames (ORF) that cannot be expressed in a gamma chain, due to a splicing defect of the pre-messenger (17, 18). The potential repertoire consists of four to six functional TRGV genes belonging to two subgroups, five TRGJ and two TRGC genes.

Polymorphisms in the number of TRGV genes (19, 20) and in the exon number of the TRGC2 gene (21) have been described in different populations and used to set up the rules for a standardized description of the CNV in IG and TR loci (5, 9). The availability of genome assemblies in different species has confirmed that the TRG locus in mammals is delimited by the *amphiphysin* (*AMPH*) gene at the 5’ end and the *STARD3 N-terminal like* (*STARD3NL*) gene at the 3′ end (5, 9). They are centromeric and telomeric, respectively, for the *Homo sapiens* locus.

A cluster organization also characterizes the small and simple rabbit TRG locus, with 11 TRGV genes upstream of two TRGJ genes and one TRGC gene in about 70 Kb, flanking by the *AMPH* and *STARD3NL* genes (22). The TRGV genes are classified in four subgroups. Also in this case, the functional repertoire is restricted to only two subgroups, the TRGV1 (with seven functions genes) and TRGV3 subgroups.

Differently, the mouse 200 kb long TRG locus comprises seven TRGV genes belonging to five subgroups, four TRGJ and four TRGC genes organized into classical V-J-C units or “TRGC cassettes”. The TRGC3 cassette is not functional because of the TRGC3 pseudogene, while the entire TRGC2 cassette is inverted in the locus with respect to the other three cassettes (IMGT Repertoire (IG and TR), https://www.imgt.org/IMGTrepertoire/> 1. Locus and genes > 2. Locus representations > TRG *Mus musculus*; 6).

The gene cassette model is also typical of the TRG locus in Cetartiodactyla and Carnivora, with the number of TRGV and TRGJ genes that varies in individual cassettes as does the number of cassettes in the diverse species (6).

The dolphin TRG locus is the smallest and simplest of all mammalian loci studied to date (23). It spans only 48 kb and includes two TRGV belonging to two distinct subgroups, three TRGJ genes and a single TRGC gene arranged in a single cassette structure.

The dromedary TRG locus spans about 105 Kb and consists of three in tandem TRGC cassettes (24). The first cassette contains the largest number of genes with five TRGV, belonging to five subgroups, and three TRGJ genes. One TRGV gene of distinct subgroups, and two TRGJ genes are in the other two cassettes.

Eighteen genes, arranged in four TRGC cassettes, called TRGC5, TRGC6, TRGC3, and TRGC4, form the pig TRG locus (25). It is in between the camelid (three cassettes) and ruminant (six or seven cassettes) loci for the number of cassettes. Five TRGV genes are in the TRGC5 cassette, and only one is in each of the other three cassettes. Two TRGJ genes compose the TRGC5 and TRGC6 cassettes, while only one TRGJ gene is present in the TRGC3 as well as in the TRGC4 cassette. The TRGV genes are assigned to seven different subgroups.

The peculiarity of the ruminant (sheep, goat and cattle) TRG locus is the presence of two paralogous loci, TRG1 and TRG2, separated by at least five chromosomal bands on the same chromosome, with the TRG2 appearing to be distinctive of these species (26, 27). As a consequence of the evolutionary TRG split, the synteny at the flanking regions of the ruminant loci has been broken, with the *AMPH* located at the 5′ end of the TRG1 locus and the *STARD3NL* gene at the 3′ end of the TRG2 locus (28). Considering both paralogous loci, a total of 14 germline TRGV genes were identified in the goat TRGs and assigned to 11 distinct subgroups. Six of 14 TRGVs are in the first cassette while three are in the TRGC3 and only one in the others. All cassettes typically contain two TRGJ genes, except for the first cassette with three TRGJ genes.

Small species-specific differences in the number of cassettes and other minor exceptions, were found in the other two ruminant species (IMGT Repertoire (IG and TR), https://www.imgt.org/IMGTrepertoire/> 1. Locus and genes > 2. Locus representations > TRG *Ovis aries*, *Bos taurus*; 6).Such a large number of TRGC cassettes but located in a single genomic region characterizes the TRG locus of carnivores (6). At present, the dog TRG locus is the largest, occupying 460 kb and comprising eight V-J-J-C cassettes (29). The reiterated cassette duplications in the canine TRG locus resulted in a total of 40 genes. There are 16 TRGV genes, more or less evenly distributed in the individual cassettes, assigned to seven subgroups, 16 TRGJ, two for each cassette, and eight TRGC genes. Only seven TRGV genes belonging to three subgroups are functional.

In this paper, the genomic organization and phylogenetic relationships of the TRG locus were investigated for the first time in two mammalian species belonging to Perissodactyla.

Our analysis showed that the TRG locus of the domestic horse (*Equus Caballus*) represents the most extensive of those so far identified in the various mammalian species, with 87 TRG genes distributed in 17 V-J-(J)-C cassettes in about 1100 Kb. The 45 TRGV genes belong to the seven mammalian TRGV subgroups (30), and used as a paradigm for the TRGV nomenclature in other species.

The TRG locus of the donkey (*Equus asinus*), which belongs to the same genus, revealed an evident similarity with the corresponding horse genomic region in terms of structure and gene sequence, even though extensive deletions that led to a reduction in the number of cassettes and genes were observed.

Furthermore, the annotation of the horse TRG genes and the availability in public databases of a transcriptome derived from splenic tissue of a healthy adult horse, allowed us to evaluate the V-J rearrangements through a clonotype analysis. This led to identify, in addition to the prominent rearrangements between TRGV and TRGJ genes from the same TRGC cassette, a consistent number of transcripts derived from a trans-cassette V-J recombination.

## 2 Results

### 2.1 The horse TRG locus

**Figure 1:**
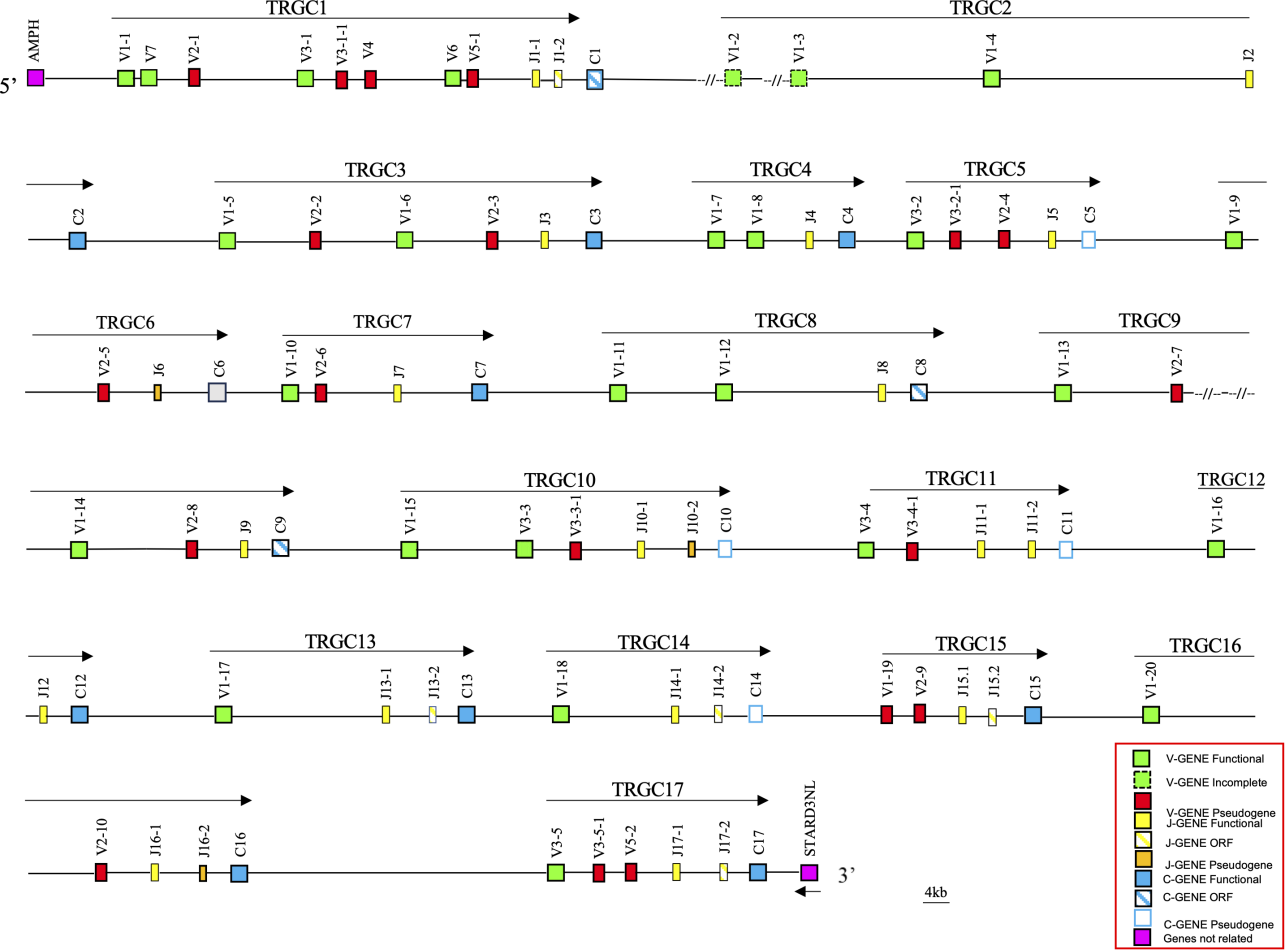
Schematic representation of the genomic organization of the horse TRG locus deduced from the EquCab3.0 genomic assembly. The name and the orientation of each TRGC cassette is indicated by an arrow. The TRGV1-2 and TRGV1-3 genes are indicated in the map with a dashed line as they are incomplete due to a gap in the genomic assembly. They are showed as functional since they were found within transcripts (see text). The diagram shows the position of all related and unrelated TRG genes according to nomenclature. The boxes representing the genes are not to scale. The vestigial TRGC6 gene is indicated by a gray box. The exons are not shown. The arrow indicates the transcriptional orientation of the *STARD3NL* gene. All gaps in the genomic sequence are indicated by the symbols (–//–).

Using the human TRG sequence as a reference, we retrieved from the whole chromosome 4 contig (GenBank ID: NC_009147) of the latest genomic assembly of the *Equus caballus* species (EquCab3.0), a region of approximately 1100 kb, corresponding to the horse TRG locus (**Figure 1**).

The recovered sequence comprises also the *AMPH* and the *STARD3NL* genes, which represent the IMGT 5’ and 3’ bornes (IMGT, https://www.imgt.org/IMGTrepertoire/LocusGenes/bornes/bornesTRG.html) since located, respectively, upstream of the first and downstream of the last gene of the TRG locus in all mammalian species in which this locus maps in a single position (6, 22–25, 29). This ensures that we have recovered the entire horse TRG genomic region.

Overall, the deduced genomic structure of the horse TRG locus reflects the organization of the TRG genes in V-J-J-C unit or TRGC “cassettes”, which is a peculiarity of artiodactyl (ruminants, pigs and camels), carnivore (dogs and cats) and mouse species (6, 25). In the horse TRG region, we identified and annotated 45 TRGV, 25 TRGJ and 17 TRGC genes distributed in 17 V-J-(J)-C cassettes, arranged in the same transcriptional orientation, which were classified from TRGC1 to TRGC17 proceeding from the 5’ to the 3’ end of the locus.

A slightly different organization characterizes the TRGC6 cassette, where a TRGJ gene (TRJ6) appeared to lie among the TRGV genes. However, a more detailed sequence analysis showed the presence of a degenerate TRGC gene between the TRGJ6-1 and TRGV1-10 genes, of which only the last two exons are recognizable.

Variations in the number of TRGV genes can be observed between cassettes, with a maximum of seven genes in the TRGC1 cassette and only one TRGV in the TRGC12, TRGC13 and TRGC14 cassettes.

The genomic position and the predicted functionality of all identified TRG genes are provided in **Supplementary Table 1**.

### 2.2 Classification and phylogenetic analysis of the horse TRGV genes

The TRGV genes were assigned to seven different subgroups by nucleotide sequence identity and their functionality predicted as defined by IMGT rules (see the Material and Methods section; IMGT Scientific chart, https://www.imgt.org/IMGTScientificChart/, accessed on 20 September 2022) (**Supplementary Table 1**). The name of the subgroups were defined according to the IMGT Nomenclature (IMGT-NC) (30). The TRGV1 subgroup is the most represented being composed of 20 genes, one of which (TRGV1-19) is a pseudogene. The TRGV1-2 and TRGV1-3 genes, although incomplete due to a gap in the genomic assembly, were considered functional as they were found within transcripts (see below paragraph 2.5). Both the TRGV2 and TRGV3 subgroups comprise ten genes, while the TRGV5 subgroup has only two genes. All TRGV2 and TRGV5 member genes are predicted to be pseudogenes. Conversely, five TRGV3 genes are functional; whereas, using the Homsap TRGVB sequence on the *Equus* genome, five hits (pseudogene sequences) were found in conserved positions, downstream of the TRGV3-1 to TRGV3-5 genes, such as the *Homsap* TRGVB gene is related to the *Homsap* TRGV10 gene. To emphasize the conserved structure, these gene sequences have been named by adding a dash and the number 1: TRGV3-1-1 to TRGV3-5-1, and their positions inserted in **Supplementary Table 1**.

Finally, both the TRGV6 and TRGV7 subgroups are formed by a single functional member gene, whereas the TRGV4 subgroup is formed by a single pseudogene.

**Figure 2:**
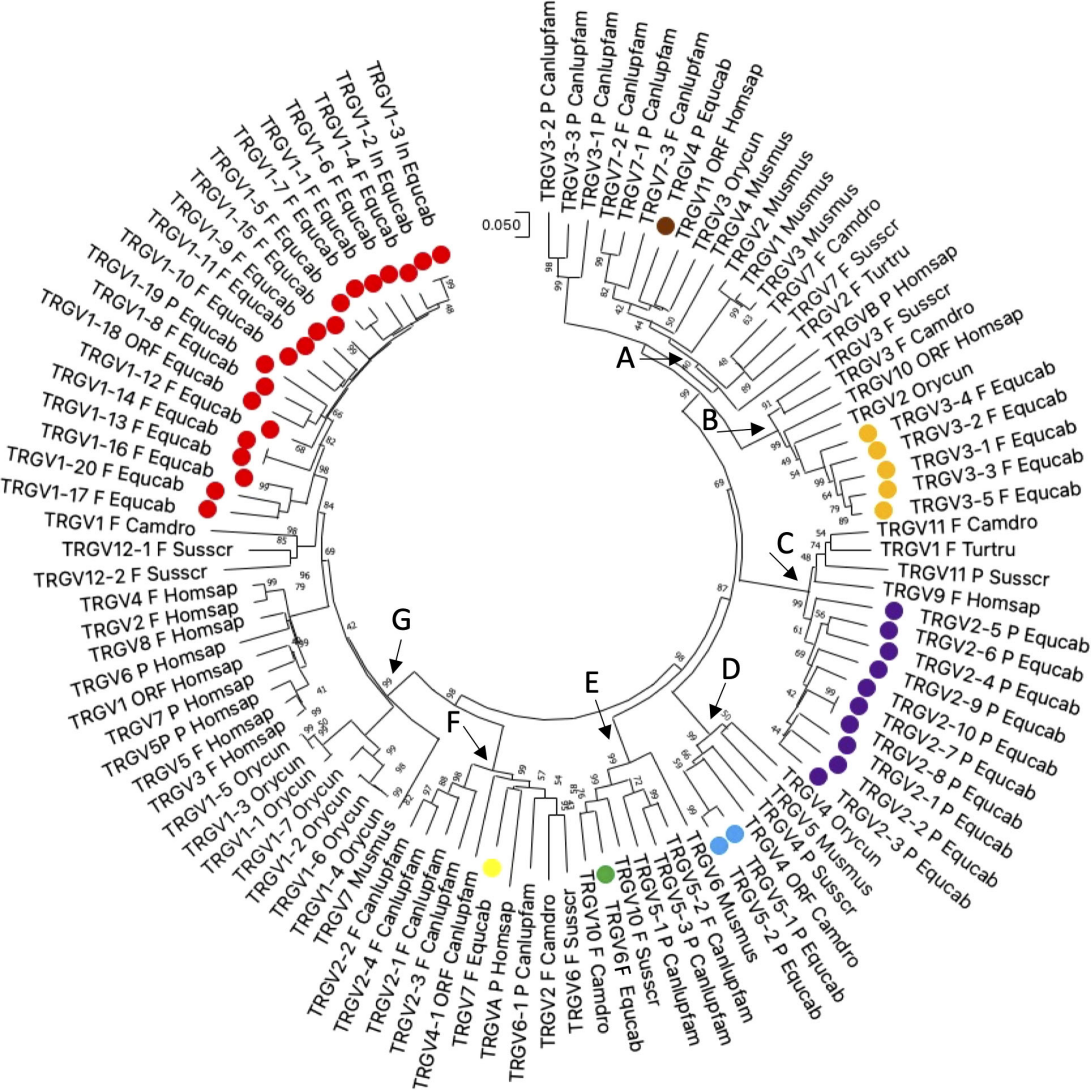
The neighbor-joining (NJ) tree inferred from the horse, dolphin, dromedary, pig, dog, rabbit, mouse and human TRGV gene sequences. The evolutionary analyses were conducted in MEGA X (33, 34). The optimal tree is shown. The tree is drawn to scale, with branch lengths in the same units as those of the evolutionary distances used to infer the phylogenetic tree. The evolutionary distances were computed using the p-distance method (35) and are in the units of the number of base differences per site. This analysis involved 103 nucleotide sequences. Codon positions included were 1st + 2nd + 3rd + Noncoding. All ambiguous positions were removed for each sequence pair (pairwise deletion option). There was a total of 410 positions in the final dataset. The horse TRGV gene are marked with coloured circles. The different colours highlight the distribution of the phylogenetic groups. The branches highlighted by the letters group mammalian genes described in the text. The gene functionality according to IMGT rules (F: functional, ORF: open reading frame, P: pseudogene) is indicated. The IMGT six-letter standardized abbreviations for species (*Equus caballus* (horse), *Homo sapiens* (human), *Mus musculus* (mouse), *Sus scrofa* (pig), *Camelus dromedarius* (dromedary), *Tursiops truncatus* (dolphin), *Oryctolagus cuniculus* (rabbit)) and nine-letter standardized abbreviation for subspecies (*Canis lupus familiaris*, dog) taxa are used.

Thus, the potential functional germline repertoire is limited to four out of seven TRGV subgroups, with TRGV1 and TRGV3 the only multi-member subgroups (**Supplementary Table 1**; **Figure 1**). It should be noted that only the TRGC1 cassette consists of TRGV genes representative of each subgroup. The deduced amino acid sequences of the potential functional germline TRGV genes and the in-frame pseudogenes are shown in **Supplementary Figure 1A**, where they are aligned according to IMGT unique numbering for the V-REGION (31) to maximize the percentage of identity. The alignment shows the heterogeneity of amino acid sequence between and within subgroups, with distinctive structural features (i.e., CDR-IMGT and FR-IMGT) of the genes for each TRGV subgroup. The TRGV1 subgroup genes present the CDR1 with 5 amino acids (except for the TRGV1-13 and TRGV1-14 genes), the CDR2 with 8 amino acids, the germline CDR3 with 5 amino acids and the FR3 with 38 amino acids (except for the TRGV1-1 gene). The TRGV3 subgroup consists of genes with the CDR1 and CDR2 of eight, the CDR3 of four and the FR3 of 39 amino acids. The TRGV7 gene structure is in between the TRGV1 and TRGV3 genes since the CDR1, the CDR2 as well as the CDR3 consist of 5, 8 and 5 amino acids respectively, while the FR3 is 39 amino acids long. Differently, the CDR1 and CDR2 of the TRGV6 gene consists respectively of nine and seven amino acids, while the CDR3 and the FR3 are four and 39 amino acids long. Finally, the TRGV2 pseudogene is made up of an eight amino acid CDR1, a seven amino acid CDR2, a five amino acid germline CDR3 and a 39 amino acids FR3. The evolutionary relationship of the horse TRGV genes was investigated by comparing all the horse gene sequences (except for the TRGV3 pseudogenes) with the corresponding gene sequences of humans, mice, rabbits, camels, dolphins, pigs and dogs, mammalian species in which the genomic organization of the TRG locus has been inferred resulting in a single chromosomal position. The V-REGION nucleotide sequences of all the selected TRGV genes were combined in the same alignment and an unrooted phylogenetic tree was constructed using the NJ method (32) (**Figure 2**). In the tree, the mammalian TRGV genes are distributed within seven clearly distinguishable groupings (A-G branches). Each branch groups corresponding genes (or gene subgroups) of the different species with a clear orthology, irrespective of their diverse genomic organization within each TRG locus, indicating their occurrence from a common ancestor. Indeed, the genes of each horse TRGV subgroup cluster with corresponding mammalian TRGV genes subgroups, when present, rather than to forming species-specific clades. Particularly, a horse TRGV subgroup is always present within every branch, which makes the horse the only species among those analyzed to possess the seven subgroups recently validated by IMGT-NC as mammalian subgroups (30).The birth-and-death evolutionary model of multigene family evolution, which explains that some duplicated genes are retained in the genome for a long time, while others are deleted or become pseudogenes, also explains the emergence of new genes that have undergone substantial diversification through species-specific duplication events, as indicated in the tree by species-specific clustering of the genes belonging to the same TRGV subgroup (the horse TRGV1, TRGV2, TRGV3 and TRGV5 genes in **Figure 2** are an example).

The horse not functional TRGV4, TRGV2 and TRGV5 subgroup genes are in the branch A, C and D, respectively; while the functional TRGV subgroup genes are in branch B (TRGV3 subgroup), E (TRGV6 subgroup), F (TRGV7 subgroup) and G (TRGV1 subgroup).

### 2.3 Classification and phylogenetic analysis of the horse TRGJ genes

25 TRGJ genes were identified along the horse TRG locus (**Figure 1**). They have been named according to the criteria for membership to the TRGC cassette and numbered for their genomics position. Eight TRGC cassettes consist of two TRGJ genes, while nine have only one TRGJ gene. **Supplementary Figure 1B** reports the nucleotide and deduced aminoacidic sequences of all TRGJ genes. 17 out of 25 were classified as functional genes (see the Material and Methods section). Five TRGJ genes were defined as ORF for a noncanonical J-motif (TRGJ13-2, TRGJ14-2, TRGJ15-2 and TRGJ17-2 genes) or heptamer sequence of the J-RS (12-RS) (TRGJ1-2 gene, the first and second nucleotides of the “cac” sequence are crucial). The remaining three TRGJ genes (TRGJ6, TRGJ10-2 and TRGJ16-2) are classified as pseudogenes for the presence of a stop codon within the J-REGION.

**Figure 3:**
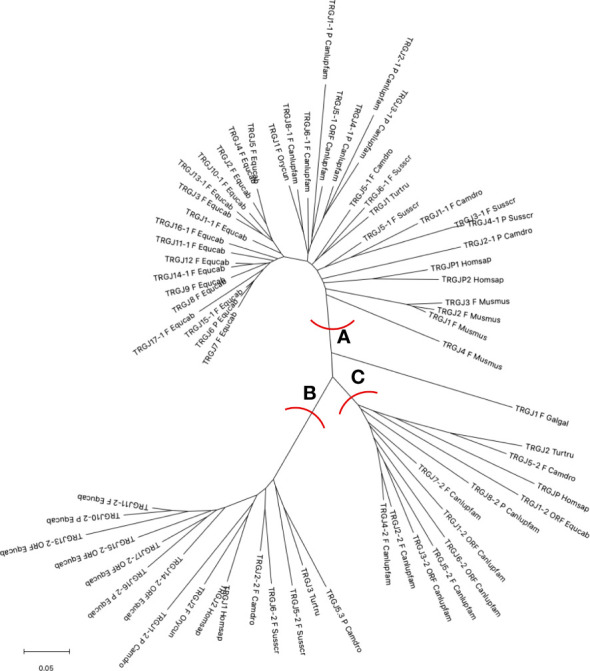
The neighbor-joining (NJ) tree inferred from the horse, dolphin, dromedary, pig, dog, rabbit, mouse and human TRGJ gene sequences. The evolutionary analyses were conducted in MEGA X (33, 34). The optimal tree is shown. The tree is drawn to scale, with branch lengths in the same units as those of the evolutionary distances used to infer the phylogenetic tree. The evolutionary distances were computed using the p-distance method (35) and are in the units of the number of base differences per site. This analysis involved 68 nucleotide sequences. Codon positions included were 1st + 2nd + 3rd + Noncoding. All ambiguous positions were removed for each sequence pair (pairwise deletion option). There was a total of 129 positions in the final dataset. The branches highlighted by the letters group mammalian genes described in the text. The gene functionality according to IMGT rules (F: functional, ORF: open reading frame, P: pseudogene) is indicated. The IMGT six-letter standardized abbreviations for species Equcab (*Equus caballus*, horse), Homsap (*Homo sapiens*, human), Musmus (*Mus musculus*, mouse), Susscr (*Sus scrofa*, pig), Camdro (*Camelus dromedarius*, dromedary), Turtur (*Tursiops truncatus*, dolphin), Orycun (*Oryctolagus cuniculus*, rabbit), Galgal (*Gallus gallus*, chicken) and nine-letter standardized abbreviation for subspecies Canlupfam (*Canis lupus familiaris*, dog) taxa are used.

The horse TRGJ genes were then aligned with the corresponding mammalian TRGJ genes for a phylogenetic analysis. The tree shows three main branches (A, B and C in **Figure 3**). Each branch contains genes of the different species. Mostly, the genes of each species form monophyletic groups, except for the cetartiodactyl species where the similarity between orthologous genes is highlighted (in A and B branches).

All horse TRGJ genes, except for the TRGJ1-2 ORF, are split into branches A and B. Branch A groups the horse TRGJ genes that, based on their physical location relative to the TRGC gene, are C-distal or are unique within its own TRGC cassette. In A, there are also either the orthologous human, dog, rabbit, dromedary, dolphin and pig genes, which, like the horse genes, occupy, within their own TRG locus, a position distal to the relative TRGC gene, and all mouse TRGJ, the pig TRGJ3-1 and TRGJ4-1, which are single genes within their own cassette. Instead, branch B groups all the TRGJ genes that are located proximally to the relative C gene of the TRGC cassette to which they belong in the TRG loci of the different species. The only exceptions are the dog C-proximal TRGJ genes located in the branch C together with the human TRGJP, dolphin TRGJ2 and dromedary TRGJ5-2, which are located in the middle of the J cluster, formed by three genes within each corresponding TRG locus.

These data highlight an evident usage pattern of the J genes based on their physical location, which play a role in the recombination process and structurally contribute to the variable domain of the receptor.

### 2.4 Classification and phylogenetic analysis of the horse TRGC genes

The horse TRGC genes exhibit a structural organization similar to that of the homologous mammalian genes (https://www.imgt.org/IMGTrepertoire/Proteins/; accessed on 25 January 2023), with a broad range of aminoacidic composition. Nine TRGC genes were predicted to be functional, while the TRGC1, TRGC8 and TRGC9 were classified as ORF, four TRGC genes, TRGC5, TRGC10, TRGC11 and TRGC14, were classified as pseudogenes and the TRGC6 is a vestigial gene (**Supplementary Table 1**).

The exon organization of the functional and ORF TRGC genes is shown in **Supplementary Figure 1C**. The first exon (EX1) of 330 pb encodes the C domain that comprises 110 amino acids; while the first part of the connecting region is encoded by different number and combination of one, two or three exons (EX2A of 60 bp, EX2B of 48 bp and EX2C of 48 bp), producing a region with a substantial amino acid diversity. The only exception is the TRGC15 gene, which lacks any EX2. The remain portion of the connecting region (CO), the transmembrane region (TM) and the cytoplasmatic region (CY) are encoded by a third exon (EX3) 137 or 143 bp long, including 45 or 47 amino acids.

Hence, the connecting region of the horse TRGC genes differed in length and amino acid sequence depending on the exons EX2. This heterogeneity is shared with the TRGC genes of other mammalian species, where the connecting region can be encoded by three exons, two exons, or only one exon (6, 21).

**Figure 4:**
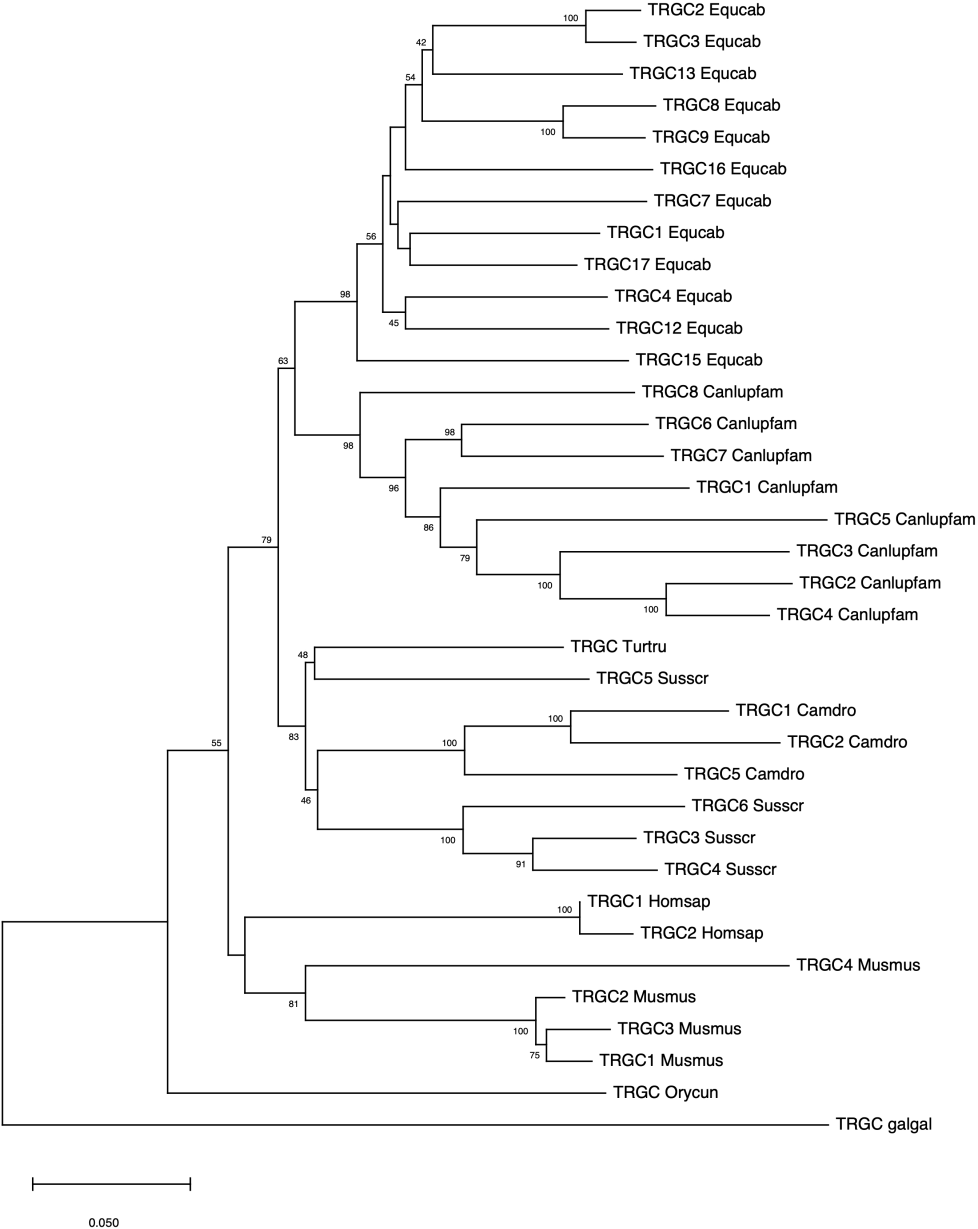
The neighbor-joining (NJ) tree inferred from the horse, dolphin, dromedary, pig, dog, rabbit, mouse and human TRGC gene sequences. The evolutionary analyses were conducted in MEGA X (33, 34). The optimal tree is shown. The tree is drawn to scale, with branch lengths in the same units as those of the evolutionary distances used to infer the phylogenetic tree. The evolutionary distances were computed using the p-distance method (35) and are in the units of the number of base differences per site. This analysis involved 36 nucleotide sequences. Codon positions included were 1st + 2nd + 3rd + Noncoding. All ambiguous positions were removed for each sequence pair (pairwise deletion option). There was a total of 707 positions in the final dataset. The IMGT six-letter standardized abbreviations for species Equcab (*Equus caballus*, horse), Homsap (*Homo sapiens*, human), Musmus (*Mus musculus*, mouse), Susscr (*Sus scrofa*, pig), Camdro(*Camelus dromedarius*, dromedary), Turtur, (*Tursiops truncatus*, dolphin), Orycun (*Oryctolagus cuniculus*, rabbit), Galgal (*Gallus gallus*, chicken) and nine-letter standardized abbreviation for subspecies Canlupfam (*Canis lupus familiaris*, dog) taxa are used.

The evolutionary relationship between the horse and the corresponding mammalian TRGC genes was also investigated. In contrast to the intermingling of the TRGV and TRGJ genes from different species, the horse TRGC genes forms a monophyletic branch as do the TRGC genes of each mammalian species/order (**Figure 4**). Hence, the tree confirms that the mammalian TRGC genes evolved in a species-specific manner, and the sequences form distinct clades consistent with the current phylogeny 33). In this regard, it should be noted the close evolutionary relationship between Perissodactyla and Carnivora.

### 2.5 Clonotype analysis

The characterization of the horse TRG genes allowed us to analyze an RNA library derived from spleen tissue available in public databases. Our aim was to evaluate the dynamics of the V-J somatic recombination process through the analysis of the CDR3 within γ chain transcripts. Since the CDR3 encompasses the juxtaposition region between the rearranged V and J genes, our analysis also provides a measure of the diversity of the γ chain repertoire.

For this purpose, each horse TRGJ germline gene sequence was used to query a transcriptome dataset derived from spleen of an adult healthy horse. Hence, all selected nucleotide sequences were translated and analyzed in detail.

We selected 363 clonotypes that are reported in **Supplementary Figure 2** grouped by the TRGJ genes and the corresponding TRGC cassette. The number of transcripts for each TRGJ gene varies considerably, from three reads for the TRGJ1-1 to 53 for the TRGJ3. This difference could be related to the level of participation of each TRGJ genes in the rearrangement. It may be possible that the transcriptome also encloses the reads of non-productive transcripts, such as sequences containing the TRGJ11-1 and TRGJ14-1 genes, which are spliced to the TRGC11 and the TRGC14 predicted pseudogenes, respectively. However, no reads were found for the TRGJ5 located upstream of the TRGC5 predicted pseudogene.

It is to be noted that we did not find sequences containing the TRGJ (both functional genes and pseudogenes) located proximal to their own TRGC genes. Therefore, a prominent participation in rearrangement for TRGJ genes distal to their own TRGC has been found. Finally, no transcripts containing the TRGJ6 gene were found. Interestingly, in all clonotypes the J region is spliced to the relevant TRGC gene. Therefore, each TRGJ germline gene is representative of the expression of its own corresponding cassette.

Consistent with the higher number of genes and their distribution in all TRGC cassettes except for the TRGC5, TRGC11 and TRGC17 ones, we identified, in 80% of the sequences (294/363), genes belonging to the TRGV1 subgroup and only 69 reads included the TRGV3 subgroup genes. No transcripts were identified containing the TRGV2, TRGV5, TRGV6 or TRGV7 subgroup genes.

In 152 out of 294 sequences, the V portion was unambiguously assigned to the corresponding TRGV1 germline genes; while, in 23 cases it was not possible to discriminate between two genes such as the TRGV1-2 or the TRGV1-3 (in 21 sequences), and the TRGV1-13 or the TRGV1-14 (in 2 sequences). However, in all cases the two alternative TRGV genes belong to the same cassette. Furthermore, in 28 of the 69 sequences comprising a TRGV3 gene, the V region was unambiguously attributed to the corresponding germline genes, whereas, in 15 sequences, two different TRGV3 genes could be assigned, namely TRGV3-3 or TRGV3-4 (in 6 sequences), and TRGV3-3 or TRGV3-5 (in 9 sequences). Unlike the TRGV1 genes, in these cases the alternative TRGV3 genes belong to different cassettes.

The determination of the V and J genes in a substantial number of transcript sequences allowed us to investigate the characteristics of the V-J rearrangement in relation to their genomic localization within the TRG locus (**Table 1**). Most TRGV genes (137/218, 62,8%) preferentially rearranged with the TRGJ genes of their own cassette. However, a consistent number of transcripts (81/218, 37,2%) derive from a trans-cassette V-J recombination. This mechanism appears most frequently to involve TRGV and TRGJ genes located in adjacent TRGC cassettes, but also it affects very distant TRGV and TRGJ genes, separated by two to a maximum of thirteen TRGC cassettes, such as the TRGV1-2 or TRGV1-3 genes in the TRGC2 cassette, and the TRGJ16-1 belonging to the TRGC16 cassette, or the TRGV3-1 gene located in the TRGC1 cassette and the TRGJ15-1 in the TRGC15 cassette. Curiously, the trans-cassette V-J recombination seems not involve the several TRGV genes located in the first TRGC cassette.

**Table 1:** Summary of the clonotype analysis.

Cassette	N° of clonotypes	Resolved*	N° of trans rearrangement	Mean CDR3 length
TRGC1	3	3	–	12,00
TRGC2	21	6	–	12,48
TRGC3	53	18	9	12,34
TRGC4	26	20	3	11,35
TRGC5	–	–	–	–
TRGC7	16	15	1	12,12
TRGC8	17	6	6	12,53
TRGC9	28	19	18	12,85
TRGC10	31	23	5	12,03
TRGC11	7	4	–	12,00
TRGC12	45	36	14	12,06
TRGC13	33	27	12	10,12
TRGC14	3	–	–	11,66
TRGC15	21	15	7	9,95
TRGC16	27	12	3	12,30
TRGC17	32	14	3	11,84
**TOTAL**	**363**	**218**	**81**	**11,84**

*indicates the number of sequences for which a germline TRGV genes has been conclusively assigned.

Bold value means to highlight the total value of the analysis.

It is evident that the somatic recombination between V and J genes belonging to different cassettes contributes to generating a large and diversified repertoire, where the same TRGV gene can recombine with different TRGJ genes and splice to the corresponding TRGC genes.

The analysis of the deduced amino acid sequences of the CDR3 loop reveal that it is heterogeneous in regard to amino acid composition and length without specific differences in relation to the TRGV or TRGJ gene usage (**Supplementary Figure 2**; **Table 1**). The mean length is 11,84 amino acids (range 5-16 amino acids).

The comparison with the germline the TRGV and TRGJ sequences allowed us also to examine the addition of random nucleotides in the junctions. Only 38 rearrangements (10%) present a direct V-J junction, without addition of nucleotides, while the most of clonotypes result in the presence of few amino acids (from one to three) in the junction due to the addition of few nucleotides (**Supplementary Figure 2**). In rare cases, the extent of change is more considerable, resulting in the presence of 4 (15 clones) or 5 (two clones) or 6 (2 clones) amino acids added in the junction region. However, the length of CDR3 is within the range. This suggests that the length of CDR3 in TR γ chain is essential for T-cell function.

### 2.6 The donkey TRG locus and comparative analysis in the *Equus* genus

**Figure 5:**
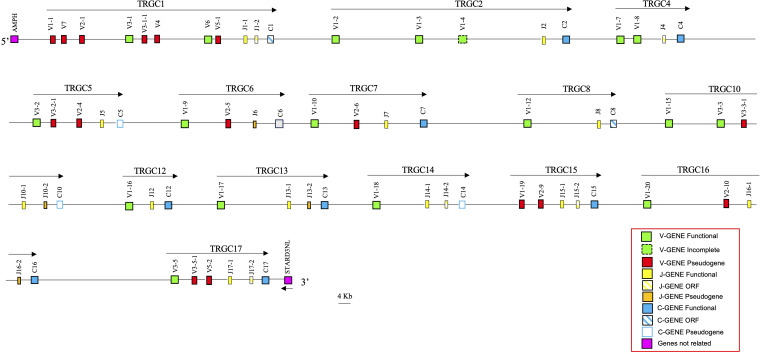
Schematic representation of the genomic organization of the donkey TRG locus deduced from the ASM1607732V2 genomic assembly. The name and the orientation of each TRGC cassette is indicated by an arrow. The diagram shows the position of all related and unrelated TRG genes according to nomenclature. The boxes representing the genes are not to scale. The vestigial TRGC6 gene is indicated by a gray box. The exons are not shown. The arrow indicates the transcriptional orientation of the *STARD3NL* gene.

Using the characterized horse TRG sequence as a reference, we retrieved from the whole chromosome 1 contig (GenBank ID: NC_052177) of the genomic assembly of the *Equus asinus* species (ASM1607732v2; 36), a region of approximately 860 kb, from the *AMPH* to *STARD3NL* genes, corresponding to the donkey TRG locus. In this region, we identified and annotated 34 TRGV, 21 TRGJ and 14 TRGC genes distributed in 14 V-J-(J)-C cassettes, arranged in the same transcriptional orientation (**Figure 5**; **Supplementary Table 1**).

A detailed structural and nucleotide comparison between the 14 donkey and the 17 horse TRGC cassettes showed that each donkey cassette matched a horse cassette perfectly. For this reason, the donkey cassettes were classified based on their homology with the horse TRGC cassettes, with the horse TRGC3, TRGC9 and TRGC11 being the three missing cassettes within the donkey locus. A perfect correspondence between the TRGV, TRGJ and TRGC genes of individual cassettes of the two species is evident, with some exceptions. Small structural differences can be observed between the TRGV and TRGJ genes of the two species.

As regards the TRGV genes, it is worth noting the lack of the TRGV1-11 gene in the donkey TRGC8 cassette as well as an incomplete TRGV1-4 in the TRGC2 cassette, where, differently, the TRGV1-2 and the TRGV1-3 genes are complete compared to the corresponding horse genes.

Therefore, as in the horse locus, the donkey TRGV1 gene subgroup is the most represented, being composed of 15 genes, two of which (TRGV1-1 and TRGV1-19) are pseudogenes. Moreover, the TRGV2, TRGV3 and TRGV5 subgroups comprise six, eight and two genes, respectively. All TRGV2 and TRGV5 member genes are predicted to be pseudogenes. Conversely, four TRGV3 genes are functional in the donkey (TRGV3-1 to TRGV3-5, with no TRGV3-4 owing to the absence of the TRGC11 cassette), and four out of the five horse TRGV3 pseudogenes were found in conserved positions, downstream of the donkey four functional TRGV3 genes, named TRGV3-1-1,TRGV3-2-1,TRGV3-3-1 and TRGV3-5-1. Finally, the TRGV7 and TRGV4 subgroups are formed by a single pseudogene, while the TRGV6 subgroup is made by a single functional gene. (**Supplementary Table 1**; **Figure 5**). Furthermore, in the donkey locus 12 out of 21 donkey TRGJ were classified as functional genes (see the Material and Methods section). Five TRGJ genes were defined as ORF for a noncanonical J-motif (TRGJ4, TRGJ14-2, TRGJ15-2 and TRGJ17-2 genes) or an anormal heptamer sequence of the J-RS (12 RS) (TRGJ1-2 gene). Four TRGJ genes are classified as pseudogenes for the presence of a stop codon within the J-REGION (TRGJ6, TRGJ10-2 and TRGJ16-2) or for an insertion (TRGJ13-2). Compared to the corresponding horse genes, the only differences consist in the functionality of the TRGJ4 gene, classified as ORF in donkey but functional in horse, and that of the TRGJ13-2, defined as pseudogene in donkey but ORF in horse.

Finally, no structural or functional differences are noted between the TRGC genes in the two species. Therefore, as in the horse locus, nine TRGC genes were predicted to be functional, while the TRGC1, the TRGC8 and the TRGC9 were classified as ORF, and the TRGC5, the TRGC10, the TRGC11 and the TRGC14 were classified as pseudogenes. Furthermore, also in this case, the TRGC6 cassette consists of a degenerate TRGC6 gene sequence.

The main characteristics and differences of the TRG genes in the two species are summarized in **Table 2**, while the genomic position and the predicted functionality of all identified donkey TRG genes are provided in **Supplementary Table 1**. It should be noted that, despite the differences, the ratio functional/total genes, remains broadly similar between the two species (**Table 2**).

**Table 2:** Comparison of the principal characteristics of the TRG locus in the two species of the *Equus* genus.

Assembly	Contig	Size (KB)	TRGV (F+P+ORF)	TRGJ (F+P+ORF)	TRGC (F+P+ORF)	Ratio F/T
*Equus caballus* EquCab3.0	NC_009147 Chromosome 4	1130	45 (26 + 19 + 0)	25 (17 + 3 + 5)	17 (9 + 5 + 3)	52/87 (0.59)
*Equus asinus* ASM1607732v2	NC_052177 Chromosome 1	850	34 (18 + 16 + 0)	21 (12 + 4 + 5)	14 (8 + 4 + 2)	38/69 (0.55)

The classification of the donkey TRG genes was validated by investigating the evolutionary relationship of the TRGV as well as the TRGC genes with the corresponding horse genes. The corresponding human TRG genes were also considered in the phylogenetic analyses.

**Figure 6:**
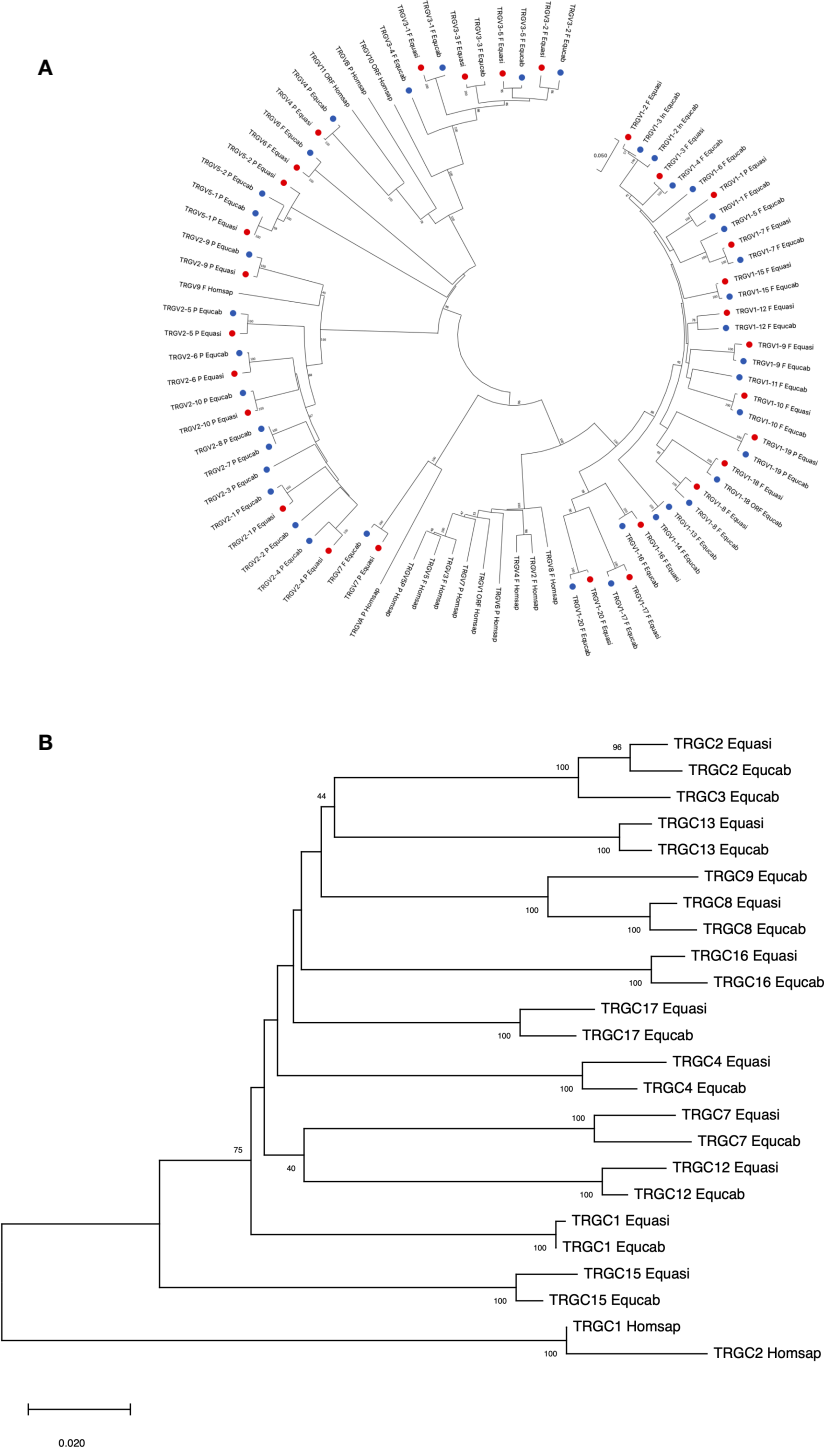
The neighbor-joining (NJ) tree inferred from the donkey, horse and human TRGV **(A)** and TRGC **(B)** gene sequences. The evolutionary analyses were conducted in MEGA X (33, 34). The optimal tree is shown. The tree is drawn to scale, with branch lengths in the same units as those of the evolutionary distances used to infer the phylogenetic tree. The evolutionary distances were computed using the p-distance method (35) and are in the units of the number of base differences per site. Codon positions included were 1st + 2nd + 3rd + Noncoding. All ambiguous positions were removed for each sequence pair (pairwise deletion option). In **(A)**, the analysis involved 83 nucleotide sequences. There was a total of 403 positions in the final dataset. The donkey and horse TRGV genes are marked with two distinct coloured circles. The phylogenetic distribution highlights the grouping of the orthologous genes of the two equid species. The gene functionality according to IMGT rules (F: functional, ORF: open reading frame, P: pseudogene) is indicated. In **(B)**, the analysis involved 24 nucleotide sequences. There was a total of 630 positions in the final dataset. The donkey and horse TRGV genes are marked with two distinct coloured circles. The IMGT six-letter standardized abbreviations for species Equasi (*Equus a*s*inus*, donkey), Equcab (*Equus caballus*, horse) and Homsap (*Homo sapiens*, human) are used.

Thus, the phylogenetic relationships of the donkey and horse TRGV genes reflect, the high grade of identity between orthologue genes, with the exception of the TRGV1-2, the TRGV1-3 and the TRGV1-4 genes because they are incomplete in one or the other of the two species (**Figure 6A**). Similarly, the TRGC genes of the two species intermingle with each other based on orthology rather than grouping in a species-specific manner, indicating that most duplication events within each TRG locus have occurred in a common ancestor (**Figure 6B**).

## 3 Discussion

Perissodactyla, an order of mammals characterized by an odd number of toes (odd-toes ungulates), contains three extant families: Tapiridae, Rhinocerotidae, and Equidae. The Equidae family consists of a single genus, *Equus*, with a variety of domestic and wild species, inhabiting a wide range of habitats and subject to different pressures from natural and artificial selection. It is a rapidly evolving mammalian family, both at the karyotype (37) and molecular (38) level and therefore represent a suitable model for evolutionary genomic studies.

In this perspective, the genes encoding the TR γ chain are an interesting example of evolutionary process among different species. Therefore, the characterization of the TRG locus in *Equus caballus* and *Equus asinus* has allowed us to investigate the equine evolution of these genes and to compare the findings with those reported in representative species of other mammalian order in an effort to improve understanding of the comparative biology of γδ T cells.

The general structure of the TRG locus in the two equid species reflects that of artiodactyls and carnivores, with a set of cassettes, each containing the basic V–J–(J)–C unit, in tandem aligned with the same transcriptional orientation, but with a relevant expansion of the number of cassettes compared to the other mammalian species.

In horse, 45 TRGV, 25 TRGJ and 17 TRGC genes, organized in 17 cassettes, span approximately 1100 Kb of the chromosome 4 contig (**Figure 1**; **Supplementary Table 1**), making the horse TRG locus the largest so far found in any mammalian species (6). In donkey, 34 TRGV, 21 TRGJ and 14 TRGC genes, organized in 14 cassettes, are distributed in a smaller region of approximately 880 kb of the chromosome 1 contig and the extension of the donkey locus still remains greater than in other mammalian species.

Until now, small differences in the genomic structure within the TRG locus have been found between species of different genus and belonging to the same subfamily, such as the presence of an extra V-J-J-C gene cassette within the goat TRG locus compared to that of sheep (28), but not between species of the same genus as in the case of the horse and donkey locus, which highlights faster mutation rates within the *Equus* genus. Consistent with our data, comparison of the genomes of five domestic horse breeds and one donkey revealed that a limited fraction of horse paralogues is not found in the donkey genome and that a significant portion of these differences involve the immunity-related and olfactory receptor genes (38).

However, the levels of genomic diversification observed between the horse and donkey loci support the hypothesis that the genome of equid species is in an ongoing evolutionary state. In line with the recent evolution of the *Equus* species (39), the donkey TRG locus, even if with three fewer V-J-(J)-C gene cassettes, retains a clear similarity to the corresponding horse genomic region in terms of gene sequence, leading us to use the same gene nomenclature. Overall, both the horse and the donkey loci retain the same percentage of potential functional genes with respect to the total, which is in line with those of dogs and cats and lower than that of the cetartiodactyl species (6).

An interesting aspect is that the potential functional germline repertoire of the horse and donkey TRGV genes is substantially constituted by two subgroups (TRGV1 and TRGV3) as in humans, rabbits and dogs, but differently than in cetartodactyls where different subgroups with one or two members form the functional repertoire of the diverse species. Since the structural differences, mainly located in CDR1, CDR2 and FR3, can be observed only between TRGV gene subgroups, it is possible to hypothesize that the extensive duplication of cassettes at the horse and donkey loci, does not correlate with the need for new genes to generate highly diverse variable domains. Or, from an evolutionary point of view, not enough time has passed yet for duplicate genes to diversify since the relative recent origin of the *Equus* genus (4.0–4.5 million years before present, Myr BP) (38, 39).

In contrast, in accordance with the “birth-and-death” model of multigene family evolution, duplicative events favored the emergence of a large number of structurally diverse TRGC genes with different exon-intron organization. Therefore, it is possible to assume that the extensive duplication of cassettes in the two species, is not related to the need for new receptors with very different variable domains, but rather to functional constant domains with biological roles perhaps related to the different functions attributed to γδ T cells (6).

However, our phylogenetic analyzes showed evolutionarily closely conserved relationships of the TRGV and TRGJ genes of equids compared to other mammalian species as a result of the strong functional pressure related to the variable region’s important role in the immune response.

Interestingly, all seven subgroups (TRGV1 to TRGV7) recently validated by the IMGT-NC (30) are present at both horse and donkey TRG loci, making the equid species a landmark for the description of TRGV subgroups in mammals.

Differently, the evolution of the TRGC genes is related to each mammalian species in line with the current phylogeny (40) in agreement with the effector role of the constant domain of the TR.

The complete annotation of the horse TRG locus also allowed us to explore the mechanism of the V-J rearrangement in this complex genomic organization, by analyzing the CDR3s of transcripts retrieved from a public splenic RNA library. Although the number of unique clones analyzed was not very high and referred to a single tissue from a single adult animal, some considerations can be made. The TRGV1 and TRGV3 subgroup genes, present simultaneously or alternately in all cassettes, are the only ones identified in the analyzed clonotypes. No transcripts containing the TRGV2, TRGV5, TRGV6 or TRGV7 subgroup genes were identified. Moreover, exclusive participation in the V-J rearrangement was found for TRGJ genes distal to its own TRGC. The most interesting result, however, is the evidence of approximately 33% trans-cassette V-J recombination. This mechanism mainly involves TRGV and TRGJ genes located in adjacent cassettes, but also affects TRGV and TRGJ genes that are far apart, expanding the diversity of the γ chain repertoire, whereby the same TRGV genes can recombine with different TRGJ genes spliced to the corresponding TRGC genes. A recent deep expression study (41) performed in different lymphoid tissues and T-cell populations described also within the pig TRG locus approximately 25% of trans-cassette V-J recombination. The increase in the percentage of V-J trans-cassette recombination found in the horse could be directly related to the higher number of cassettes present in the equid locus.

In conclusion, our data and the comparative analysis are in agreement with the evolutionary idea of the existence of a *Equus* recent common ancestor and a family (Equidae) in continuous and rapid evolution, for which a mutation rate between horses and donkeys is more comparable to that between species belonging to different genera (see goat and sheep) rather than to species of the same genus.

## 4 Materials and methods

### 4.1 Horse and donkey genome analysis

To determine the horse TRG locus location, the EquCab3.0 genome sequence (GenBank accession: GCA_002863925.1), was searched using the BLAST algorithm. A sequence of 1.092.921 bp was retrieved directly from the reference sequence NC_009147 (*Equus caballus* chromosome 4 genomic sequence) available at NCBI from 9858268 to 8765347 (complement) positions. Particularly, the analyzed region extends from the *AMPH* (pos: 9867972-10095199) to the *STARD3NL* (pos 8714273-8761799) genes, found flanking the TRG locus of most mammalian species (https://www.imgt.org/IMGTrepertoire/LocusGenes/#h1_6, accessed on 20 September 2022).

The donkey TRG locus was retrieved from the reference sequence NC_052177 (*Equus asinus* chromosome 1 genomic sequence) of the ASM1607732V2 genome assembly (GenBank accession GCA_016077325.2). The locus is 858.484 bp long from 95349412 to 94490928 positions (complement) and is flanking by the *AMPH* (pos: 95,359,239-95,586,025**)** and the *STARD3NL* (pos 94,436,282-94,487,656) genes.

All horse TRG genes within the genome sequence were identified and annotated using the available human TRG genomic sequences (NC_000007.14 pos: 38220024-38388055) as a reference. All donkey TRG genes within the genome sequence were identified and annotated using the horse TRG genomic sequences as a reference. The beginning and end of each coding exon were identified with accuracy by the presence of splice sites or flanking recombination signal (RS) sequences of the V and J genes.

The locations of the horse and donkey TRG genes are provided in **Supplementary Table 1**.

### 4.2 Classification of the TRG genes

The functionality of the TRGV genes was predicted through the manual alignment of sequences adopting the following parameters: (a) identification of the leader sequence at the 5′ of the V genes; (b) determination of proper RS sequences located at 3′ of the V (V–RS); (c) determination of conserved acceptor and donor splicing sites; (d) estimation of the expected length of the coding regions; (e) absence of frameshifts and stop codons in the coding regions of the genes. Conversely, a germline gene is qualified as ORF (open reading frame) if the coding region has an open reading frame, but alterations have been described in the splicing sites and/or RS sequences, and/or in changes of conserved amino acids. Finally, a germline gene is qualified as a pseudogene (P) if its coding region has stop codon(s) and/or frameshift mutation(s).

The horse genes were classified and annotated first. The horse TRGV genes were grouped in different subgroups based on the percentage of nucleotide identity by using the Clustal Omega alignment tool, which is available at the EMBL-EBI website (http://www.ebi.ac.uk/, accessed on 20 September 2022), adopting the criterion that sequences with a nucleotide identity of more than 75% in the coding region of a TR V gene (i.e., L-PART1+V-EXON) belong to the same subgroup (14). Subsequently, the name of each subgroup was defined based on the recent validation of the TRGV subgroups in mammals by the IMGT Nomenclature Committee (30).

The horse TRGJ genes were named by a number in accordance with the name of the belonging TRGC cassette, followed by a hyphen and a number corresponding to their position within the cassette. The functionality of the TRGJ genes was predicted based on the: a) determination of proper 12 RS sequences at the 5′, b) determination of conserved acceptor splicing sites at the 3’ end, c) absence of frameshifts and stop codons in the coding regions, d) conservation of the canonical FGXG amino acid J-motif.

The horse TRGC genes, numbered (from TRGC1 to TRGC17) on the basis of their location from 5’ to 3’ end in the locus, define the name of the cassettes. Nine TRGC genes were predicted to be functional, while the TRGC1, TRGC8 and TRGC9 genes were defined as ORF for the lack of the stop codon (TRGC1) and because of 2nd CYS is missing (TRGC8 and TRGC9). Finally, TRGC5, TRGC6, TRGC10, TRGC11 and TRGC14 genes were classified as pseudogenes due to an abnormal structure. All donkey genes were classified and annotated on the basis of their homology to horse genes.

The position and the predicted functionality of horse and donkey TRG genes are reported in **Supplementary Table 1**.

### 4.3 Phylogenetic analysis

The human, mouse, rabbit, dolphin and dog TRGV, TRGJ and TRGC gene sequences used for the phylogenetic analysis, as annotated, were retrieved from the IMGT^®^ (IMGT Repertoire (IG and TR), https://www.imgt.org/IMGTrepertoire/> 1. Locus and genes, accessed on 20 September 2022), IMGT/GENE-DB (42), The dromedary and pig gene sequences were retrieved from the GenBank database with the following accession numbers: GCA_000803125.1, JN165102, and JN172913 (dromedary TRG locus as characterized by Antonacci et al. (24)); and NC 010451 (pig TRG locus as characterized by Linguiti et al. (25)).

For the horse phylogenetic analysis, we combined the nucleotide sequences of all V-REGION of the horse TRGV genes (except for the TRGV3 pseudogenes) with the corresponding gene sequences of humans, mouse, rabbits, dolphins, dogs, dromedaries and pigs. All functional genes, ORFs and pseudogenes (excepted for the dog TRGV1-1) were selected.

Similarly, the coding region sequences of the horse TRGJ as well as TRGC (only functional and ORF) genes were aligned with the corresponding gene sequences of the same mammalian species. The corresponding TRGJ1 and TRGC sequences (43) from *Gallus gallus* were used as outgroup.

In the same way, the donkey TRGV as well as TRGC gene sequences were combined with the corresponding horse and human gene sequences for an evolutionary analysis.

Multiple alignments of the gene sequences under analysis were carried out with the MUSCLE program (44). The evolutionary analyses were conducted in MEGA X (33, 34). We used the neighbor-joining (NJ) method to reconstruct the phylogenetic tree (32). The evolutionary distances were computed using the p-distance method (35) and are in the units of the number of base differences per site.

### 4.4 Horse transcriptome analysis

An RNA-seq transcriptomic dataset derived from spleen of an adult healthy horse and available at the NCBI Sequencing Read Archive (SRA, ID: ERX2600993) was examined to identify distinct TRG clonotypes. All the 25 horse TRGJ germline gene sequences were used to analyze the transcriptome data and to create datasets distinct for each TRGJ gene, considering only sequences with a percentage of nucleotide identity from 98 to 100%. The resulting reads of each dataset were then translated and only the unique in frame sequence with a complete CDR3 were analyzed in detail. We obtained an output comprising continuous sequences of an average length of 120 bp, each containing the 3’part of the TRGV region, any no-templated bases, the entire TRGJ region and the 5’ part of the TRGC region. Therefore, all reads included the V-gamma CDR3-IMGT, defined as the amino acid stretch 105-117, starting at the codon after the last cysteine (2nd-CYS 104) of the TRGV gene and ending at the amino acid before the phenylalanine (J-PHE 118) in the conserved motif FGXG of TRGJ genes.

We aligned each transcript with the germline TRG gene sequences to attribute corresponding TRGV and TRGC genes. While the TRGJ and TRGC portion were matched in all cases to the corresponding germline genes, the high degree of nucleotide identity, especially between genes belonging to the same subgroup, and the replacement of the deleted TRGV ends with no-templated bases, has often made it impossible to assign a unique TRGV gene to every clonotype. However, each TRGV gene has been assigned to its own subgroup based on the different sequence in the germline CDR3 among TRGV subgroups.

## Data availability statement

The original contributions presented in the study are included in the article/**Supplementary Material**. Further inquiries can be directed to the corresponding author.

## Author contributions

SM: Conceptualization, Writing – original draft, Writing –review & editing. FG: Conceptualization, Methodology. NP: Methodology. AP: Data curation. AJ: Methodology. RA: Conceptualization, Writing – original draft, Writing – review & editing.
