# Supplementary material for: Genomic and comparative analysis of the T cell receptor gamma locus in two Equus species
Source: Front Immunol. 2023 Sep 15;14:1264949. doi: 10.3389/fimmu.2023.1264949 (PMC10540303; doi:10.3389/fimmu.2023.1264949)
Supplement: Supplementary file 3 [file DataSheet_3.doc]

**Reference cited only in the Supplementary Material**

Lefranc M-P, Pommié C, Kaas Q, Duprat E, Bosc N, Guiraudou D, et al. IMGT unique numbering for immunoglobulin and T cell receptor constant domains and Ig superfamily C-like domains. Dev Comp Immunol (2005) 29:185–203. doi: 10.1016/j.dci.2004.07.003
