# Supplementary material for: Genomic and comparative analysis of the T cell receptor gamma locus in two Equus species
Source: Front Immunol. 2023 Sep 15;14:1264949. doi: 10.3389/fimmu.2023.1264949 (PMC10540303; doi:10.3389/fimmu.2023.1264949)
Supplement: Supplementary file 4 [file Table_1.pdf]

Supplementary Table 1. Description of the TRG genes in the *Equus caballus* chromosome 4 genome assembly (NCBI Reference Sequence NC_009147) and in *Equus asinus* chromosome 1 genome assembly (NCBI Reference Sequence NC_052177). The position of all genes and their classification and functionality are reported.

		Equus caballus		*Equus asinus*	
**TRGC cassette**	**Gene classification**	**Functionality**	**Position**[a] (complement)	**Functionality**	**Position**[a] (complement)
TRGC1	TRGV1-1	F	9857808-9858268	P[2]	95348951-95349412
	TRGV7	F	9855810-9856249	P[2]	95346941-95347381
	TRGV2-1	P[0]	9850802-9851273	P[0]	95341914-95342385
	TRGV3-1	F	9836125-9836594	F	95327246-95327715
	TRGV3-1-1	P[1]	9832600-9832842	P[1]	95323712-95323955
	TRGV4	P[1]	9828978-9829284	P[1]	95320084-95320391
	TRGV6	F	9816328-9816909	F	95307435-95308016
	TRGV5-1	P[2]	9814593-9815030	P[2]	95305658-95306099
	TRGJ1-1	F	9806519-9806578	F	95297812-95297871
	TRGJ1-2	ORF[5]	9803883-9803937	ORF[5]	95295176-95295230
	TRGC1	ORF[8]	9794742-9800611	ORF[8]	95286029-95291904
TRGC2	TRGV1-2	Inc*	9775788-9776174	F	95266070-95266532
	TRGV1-3	Inc*	9768491-9768583	F	95239666-95240129
	TRGV1-4	F	9741455-9741918	Inc*	95221326-95221368
	TRGJ2	F	9704004-9704063	F	95201571-95201630
	TRGC2	F	9686238-9698198	F	95183797-95195749
TRGC3	TRGV1-5	F	9665793-9666255	-	-
	TRGV2-2	P[3]	9654112-9654843	-	-
	TRGV1-6	F	9642510-9642973	-	-
	TRGV2-3	P[3]	9631198-9631671	-	-
	TRGJ3	F	9624601-9624660	-	-
	TRGC3	F	9605210-9618811	-	-
TRGC4	TRGV1-7	F	9588343-9588805	F	95167152-95167615
	TRGV1-8	F	9584590-9585050	F	95163325-95163785
	TRGJ4	F	9577806-9577865	ORF[7]	95156541-95156600
	TRGC4	F	9564721-9573718	F	95144103-95152484
TRGC5	TRGV3-2	F	9532182-9532651	F	95111532-95112000
	TRGV3-2-1	P[1]	9528148-9528284	P[1]	95107450-95107586
	TRGV2-4	P[3]	9520897-9521369	P[3]	95098978-95099450
	TRGJ5	F	9514566-9514625	F	95092669-95092728
	TRGC5	P[9]	9502067-9510574	P[9]	95080828-95088577

TRGC6	TRGV1-9	F	9482242-9482702	F	95061019-95061479
	TRGV2-5	P[3]	9468316-9468787	P[3]	95047099-95047570
	TRGJ6	P[6]	9460613-9460672	P[6]	95039569-95039628
	TRGC6	vg[#]	9451533-9453869	vg[#]	95030459-95032797
TRGC7	TRGV1-10	F	9441712-9442175	F	95020614-95021077
	TRGV2-6	P[3]	9428892-9429355	P[3]	95007870-95008333
	TRGJ7	F	9419007-9419066	F	94999001-94999060
	TRGC7	F	9392335-9408026	F	94969584-94987984
TRGC8	TRGV1-11	F	9373474-9373940	-	-
	TRGV1-12	F	9359213-9359676	F	94935475-94935938
	TRGJ8	F	9336992-9337051	F	94913005-94913064
	TRGC8	ORF[8]	9328532-9333308	ORF[8]	94904496-94909289
TRGC9	TRGV1-13	F	9308255-9308720	-	-
	TRGV2-7	P[3]	9293124-9293593	-	-
	TRGV1-14	F	9275928-9276393	-	-
	TRGV2-8	P[3]	9260796-9261265	-	-
	TRGJ9	F	9253715-9253774	-	-
	TRGC9	ORF[8]	9245268-9250056	-	-
TRGC10	TRGV1-15	F	9228404-9228866	F	94887603-94888065
	TRGV3-3	F	9212713-9213181	F	94871879-94872348
	TRGV3-3-1	P[1]	9208303-9208548	P[1]	94867470-94867713
	TRGJ10-1	F	9197009-9197068	F	94856165-94856224
	TRGJ10-2	P[6]	9190517-9190566	P[6]	94849500-94849549
	TRGC10	P[9]	9166552-9187024	P[9]	94832198-94846090
TRGC11	TRGV3-4	F	9146752-9147220	-	-
	TRGV3-4-1	P[1]	9142104-9142334	-	-
	TRGJ11-1	F	9131170-9131229	-	-
	TRGJ11-2	F	9124103-9124152	-	-
	TRGC11	P[9]	9106857-9120691	-	-
TRGC12	TRGV1-16	F	9085928-9086391	F	94809353-94809816
	TRGJ12	F	9078994-9079053	F	94802446-94802505
	TRGC12	F	9067775-9074999	F	94790987-94798446
TRGC13	TRGV1-17	F	9048297-9048763	F	94773744-94774210
	TRGJ13-1	F	9025525-9025579	F	94751534-94751588
	TRGJ13-2	ORF[7]	9019338-9019387	P[3]	94745376-94745426
	TRGC13	F	9010168-9015967	F	94736210-94742006
TRGC14	TRGV1-18	F	8997295-8997759	F	94720637-94721101
	TRGJ14-1	F	8981689-8981748	F	94705072-94705131
	TRGJ14-2	ORF[7]	8976097-8976146	ORF[7]	94699467-94699517
	TRGC14	P[10]	8946384-8972493	P[10]	94671249-94695873

TRGC15	TRGV1-19	P[4]	8928033-8928495	P[4]	94652941-94653396
	TRGV2-9	P[3]	8923317-8923874	P[3]	94648224-94648781
	TRGJ15-1	F	8917561-8917618	F	94642468-94642525
	TRGJ15-2	ORF[7]	8913256-8913305	ORF[7]	94638163-94638218
	TRGC15	F	8907109-8909076	F	94632267-94634233
TRGC16	TRGV1-20	F	8891262-8891736	F	94616409-94616883
	TRGV2-10	P[3]	8866444-8866919	P[3]	94591464-94591939
	TRGJ16-1	F	8859634-8859693	F	94585241-94585300
	TRGJ16-2	P[6]	8853004-8853055	P[6]	94578615-94578666
	TRGC16	F	8842475-8849091	F	94568079-94574706
TRGC17	TRGV3-5	F	8797106-8797574	F	94522652-94523120
	TRGV3-5-1	P[1]	8792970-8793213	P[1]	94518512-94518754
	TRGV5-2	P[2]	8787440-8787879	P[2]	94512983-94513395
	TRGJ17-1	F	8781784-8781843	F	94507354-94507413
	TRGJ17-2	ORF[7]	8775153-8775202	ORF[7]	94500728-94500777
	TRGC17	F	8765347-8771971	F	94490928-94497537

[a] L-PART1/ V-exon for TRGV genes;
[*]Inc: incomplete;
[#]vg: vestigial
[0]Stop codon in L-PART1; Indel in V-exon
[1]No L-PART1; Indel in V-exon
[2]Indel and stop codon in V-exon
[3]Indel in coding region
[4]Stop codon in V exon
[5]noncanonical J-HEPTAMER
[6]Stop codon in J-REGION
[7]No canonic J-MOTIF
[8]No stop codon
[9]Del in EX3
[10]Noncanonical SPLICE in EX2C
